# Vaping prevention curriculum in India

**DOI:** 10.3389/fpubh.2025.1686030

**Published:** 2025-10-28

**Authors:** Vaibhav Sahni, Abhishek Shankar

**Affiliations:** All India Institute of Medical Sciences New Delhi, New Delhi, India

**Keywords:** vaping, Electronic Nicotine Delivery Systems (ENDS), curriculum, prevention, India, education

As of 2022, 23.9% of the Indian population (aged 15 years and above) uses tobacco (10.4% females and 36.8% males) (1). Further, 4.6% of the Indian population (≥15 years of age) smokes cigarettes as per 2022 data (0.4% females and 8.6% males) (1).

As per data from the National Family Health Survey-5 (NFHS-5), 2019–2021, from 47,343 adolescent (15–24 years of age) participants (42,254 females and 5,089 males), 1.3% females and 20.3% males were tobacco users (2). Males were found to be tobacco users more commonly as compared to females in both the young adult and late adolescent age groups: 31.8%, 2.2% and 15.9%, 1.2%, respectively (2).

According to data from 2021, tobacco was implicated in 18% or about 274,000 ischemic heart disease (IHD) mortalities, 60.4% (33,500) of all deaths from lung cancer, 14.2% (95,200) deaths from stroke and 40.7% (362,700) mortalities from Chronic Obstructive Pulmonary Disease (COPD) (1). In 2021, tobacco was responsible for the loss approximately 12.6% of the total Disability Adjusted Life Years (DALYs) which is a loss of around 28.9 million DALYs in itself. Tobacco was responsible for 887,300 lung cancer DALYs, 7.8 million IHD DALYs, 7.9 million COPD DALYs, and 2.6 million stroke DALYs (1).

In apparent public health interest, Electronic Nicotine Delivery Systems (ENDS) are banned in India (1). The rationale behind this policy has been stated to revolve around issues such as nicotine addiction, harmful additives, possible carcinogens, waste disposal, and manufacturing-related environmental hazards (3).

Despite the ban, ENDS continue to be available via illicit sources in India and readily so to be bought and used by consumers with vendors in probably all cases not being bothered to ascertain the age of the individuals buying these products.

Based on some evidence e-cigarettes are used as a smoking cessation intervention considering their safety profile in comparison to conventional cigarettes but the possibility of dual addiction cannot be ignored especially in the context of low-middle-income countries (4).

A growing concern surrounds the usage of these devices by people who were not smoking conventional cigarettes in the first place and indeed by adolescents and young adults who might be at risk of falling into nicotine addiction, which could then serve as a gateway to tobacco consumption and other substance abuse behavior (5).

A rampant unregulated gray/black market can possibly be filled with dubious and dangerous products which can cause significant harm to users such has been reported with the E-cigarette- or Vaping-Use-Associated Lung Injury outbreak in the US as well as incidents of modified devices causing explosive injuries (6, 7). In light of the situation described, it becomes imperative to educate as many people as possible, particularly those in younger age groups who might be more impressionable, regarding these devices and their risk profile (8).

A significant step forward in this regard will be to incorporate vaping prevention information into school curricula. This can be accomplished by adding this section alongside information on tobacco and/or other substance abuse-related topics. Individuals at a young and possibly impressionable age are likely to benefit from scientifically correct information when this is provided in a formal manner from a perceptible reliable source and individual (teacher).

School-based interventions, such as the Vaping: know the Truth curriculum, has been observed to be successful in increasing vaping harm-related knowledge among the youth (9). This intervention was innovative in terms of being peer-to-peer and online while also providing guidance on ways to quit vaping (9).

The CATCH My Breath program was found to be effective, feasible, and well-received when administered to 6,217 pupils over 25 high- and middle-schools across a 4-year time period in eight counties in the Appalachian region of the United States (10).

The OurFutures Vaping Program based out of Australia is another prevention-based initiative targeted toward adolescents (11).

The American Academy of Pediatrics also offers a curriculum aimed toward youth vaping and e-cigarette cessation and prevention (12).

There is however, a lack of such programs reported from the Southeast Asia region. Ideally, the strategy should be region-specific, however, existing programs from other parts of the world can be adapted in part or as a whole to evaluate their effectiveness, as a starting point.

A survey of medical students from Scotland (*n* = 606 comprising about 12% of all Scottish medical students) found that a vast majority (95%) reported e-cigarettes to not being covered sufficiently in their curriculum while 61% admitted that there was no mention of e-cigarettes in their coursework (13). Most respondents (98%) were not aware of the availability of any cessation services (13).

Previous work has highlighted shortcomings in the knowledge of dental students in the US and Spain to deal with a rise in e-cigarette usage and imparting relevant information to patients in this regard (14). This study recommended educational programs and incorporation of information on the hazards of e-cigarette usage in dental curricula (14).

The ill-effects of tobacco usage are taught in medical and dental schools in India, however, previous research has highlighted the largely didactic nature of instruction and a need to integrate tobacco counseling training not just in India but across curricula the world over (15).

There is room in Indian academic curricula to add information on ENDS in the MBBS (Bachelor of Medicine, Bachelor of Surgery), BDS (Bachelor of Dental Surgery), B.Sc. Nursing and B.Pharm courses (16–19). Healthcare professionals will probably be the first contacts of many ENDS-using individuals reporting to clinics and should be trained in identifying such individuals and counseling them appropriately. Not only this, healthcare professionals may largely be perceived as reliable sources of information and it is only when these individuals themselves have appropriate information on ENDS can they provide accurate information further.

The vaping prevention curricula at the school-level can be focused on equipping students with knowledge regarding the harmful effects of using these devices and help device refusal strategies. At the university-level, ENDS education can delve into greater scientific detail regarding the risk profile of vaping along with integrating cessation counseling skills. The delivery methods for a vaping prevention curriculum can be both online and in-person, with ENDS education at the university-level including a practical component to integrate cessation counseling skills.

**Figure 1:**
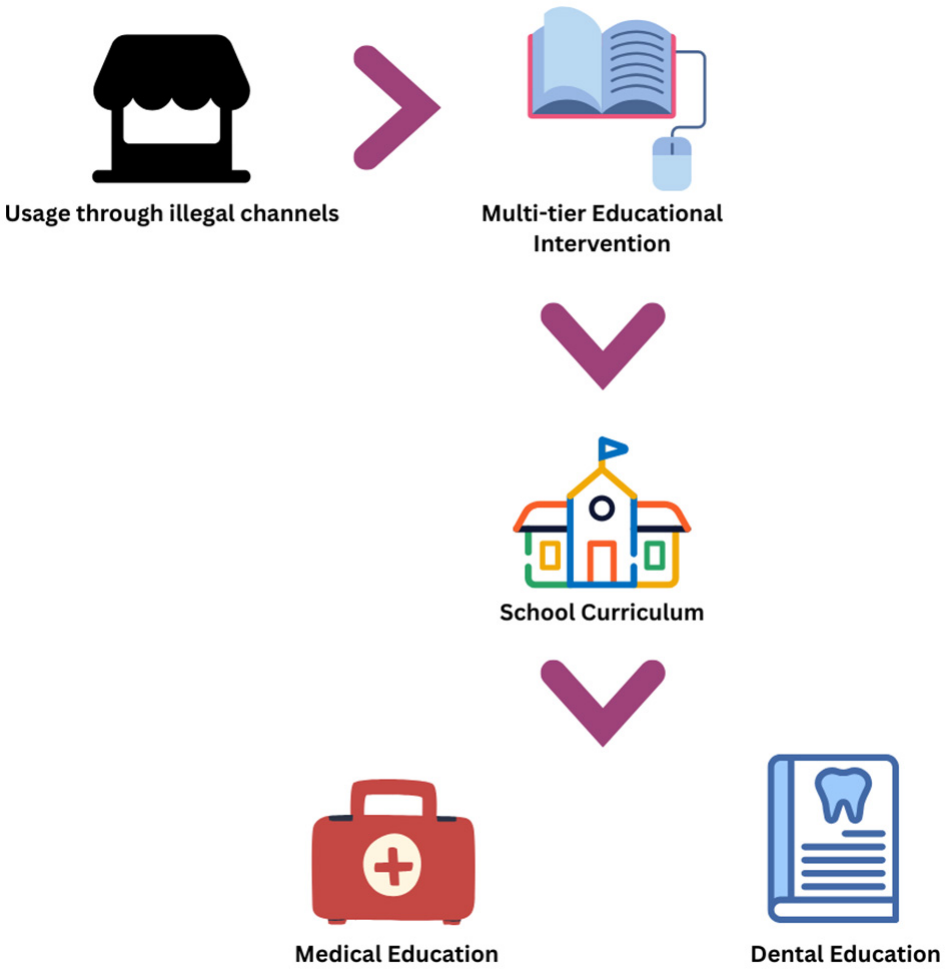
A workflow of the ENDS situation and suggested educational intervention for India.

Figure 1 illustrates a workflow of the situation and suggestions described.
